# Neuromuscular Electrical Stimulation as a Method to Maximize the Beneficial Effects of Muscle Stem Cells Transplanted into Dystrophic Skeletal Muscle

**DOI:** 10.1371/journal.pone.0054922

**Published:** 2013-03-19

**Authors:** Giovanna Distefano, Ricardo Jose Ferrari, Christopher Weiss, Bridget M. Deasy, Michael L. Boninger, G. Kelley Fitzgerald, Johnny Huard, Fabrisia Ambrosio

**Affiliations:** 1 Department of Physical Medicine and Rehabilitation, University of Pittsburgh, Pittsburgh, Pennsylvania, United States of America; 2 Department of Physical Therapy, University of Pittsburgh, Pittsburgh, Pennsylvania, United States of America; 3 Department of Orthopaedic Surgery, University of Pittsburgh, Pittsburgh, Pennsylvania, United States of America; 4 Department of Microbiology and Molecular Genetics, University of Pittsburgh, Pittsburgh, Pennsylvania, United States of America; 5 McGowan Institute for Regenerative Medicine, University of Pittsburgh, Pittsburgh, Pennsylvania, United States of America; University of Rome La Sapienza, Italy

## Abstract

Cellular therapy is a potential approach to improve the regenerative capacity of damaged or diseased skeletal muscle. However, its clinical use has often been limited by impaired donor cell survival, proliferation and differentiation following transplantation. Additionally, functional improvements after transplantation are all-too-often negligible. Because the host microenvironment plays an important role in the fate of transplanted cells, methods to modulate the microenvironment and guide donor cell behavior are warranted. The purpose of this study was to investigate whether the use of neuromuscular electrical stimulation (NMES) for 1 or 4 weeks following muscle-derived stem cell (MDSC) transplantation into dystrophic skeletal muscle can modulate the fate of donor cells and enhance their contribution to muscle regeneration and functional improvements. Animals submitted to 4 weeks of NMES after transplantation demonstrated a 2-fold increase in the number of dystrophin+ myofibers as compared to control transplanted muscles. These findings were concomitant with an increased vascularity in the MDSC+NMES group when compared to non-stimulated counterparts. Additionally, animals subjected to NMES (with or without MDSC transplantation) presented an increased maximal specific tetanic force when compared to controls. Although cell transplantation and/or the use of NMES resulted in no changes in fatigue resistance, the combination of both MDSC transplantation and NMES resulted in a faster recovery from fatigue, when compared to non-injected and non-stimulated counterparts. We conclude that NMES is a viable method to improve MDSC engraftment, enhance dystrophic muscle strength, and, in combination with MDSC transplantation, improve recovery from fatigue. These findings suggest that NMES may be a clinically-relevant adjunct approach for cell transplantation into skeletal muscle.

## Introduction

Cellular therapy is a promising method to enhance skeletal muscle regeneration after injury or disease. However, the use of cell therapies for myopathies has often been limited by impaired donor cell survival [1] and limited functional improvements following transplantation [2]. An essential role of the muscle microenvironment on donor cell fate determination has been suggested [3]–[5], and it may be argued that the success of cellular therapies is reliant on the characteristics of the host tissue into which the cells are introduced [6]. Therefore, a challenge in the clinical translation of cellular therapies is to develop reasonable adjunct approaches that modulate the host microenvironment, or niche, and maximize functional benefits following cell transplantation. The development of clinically relevant approaches to modulate the niche and optimize cell transplantation is, therefore, important.

Recent studies realized in our laboratory have shown that motor unit activation through mechanical stimulation protocols, such as functional overloading [7] and treadmill running [8], results in improved vascularity and increased myogenic potential of donor MDSCs. Similarly, an increased contribution of systemically-administered bone-marrow derived stem cells (BMDCs) to the injured muscle has been observed when the muscle was subjected to treadmill running [9]. Other exercise modalities, such as swimming, have also demonstrated an enhanced engraftment of transplanted cells [10]. Taken together, these studies suggest that donor stem cells respond to the skeletal muscle needs for regeneration and/or repair that result from injury or overuse of the host.

Neuromuscular Electrical Stimulation (NMES) is a common, inexpensive and safe modality used by physical therapists to stimulate muscular adaptations, promote angiogenesis, and increase muscle strength. It also allows for a targeted administration and physiological response of the specific muscle of interest. The well-documented ability of NMES to up-regulate VEGF expression [11], [12] further suggests that this modality is a feasible method to modulate the host microenvironment and donor cell behavior in cases of muscle injury or pathology.

The purpose of this study was to evaluate the ability of NMES to modulate the fate of donor cells and enhance their contribution to muscle regeneration and functional improvements when transplanted into dystrophic skeletal muscle. While we anticipate the transplantation alone of wild type MDSC will result in limited functional benefits of the host muscle, we hypothesize that the application of NMES will enhance the myogenic potential of MDSCs and result in an improved functional capacity.

## Materials and Methods

### MDSC isolation from wild type and dystrophic mice

MDSCs were isolated from skeletal muscle samples obtained from female 3-week old wild type (C57BL/6J; The Jackson laboratory, Bar Harbor, ME, USA) mice using a previously described modified pre-plate technique [13].

Briefly, harvested tissues were immediately placed in saline solution and finely minced in 1× Hank's Balanced Salt Solution (HBSS; Invitrogen, 24020). Minced pieces were subsequently digested using 0.2% Collagenase, Dispase (2.4 units/mL HBSS), and 0.1% Trypsin. After digestion, the tissue/HBSS solution was passed through needles ranging in size in order to dissociate any larger cellular pieces. The solution was then placed into a 25 cm^2^ collagen-coated culture plate for 2 hours, and transferred to another 25 cm^2^ coated culture plate for 24 hours. This step was repeated an additional four times. The 6^th^ pre-plate population of cells has been identified as displaying myogenic potential and immune-privileged behavior [14].

MDSCs were cultured in normal growth medium, consisting of Dulbecco's Modified Eagle Medium (Invitrogen, 11995-073) supplemented with 10% fetal bovine serum, 10% horse serum, 1% penicillin/streptomycin, and 0.5% chick embryo extract (Gibco-BRL). The cells were maintained in growth medium to approximately 30% confluence and were subsequently passaged.

### Animal care and groups

A total of 36 male dystrophic (*mdx*) mice with severe combined immunodeficiency (SCID) (3–5 months old) were used. *Mdx* mice lack the protein dystrophin and demonstrate chronic cycles of active degeneration/regeneration [15], making it a useful model of Duchenne Muscular Dystrophy to better understand the effects of host muscle niche on donor stem cell fate. SCID animals were used in order to minimize the immune rejection against transplanted MDSC surface antigens.

Mice were housed 4 per cage in a room kept at 20–23°C and a 12:12 h dark-light cycle. Animals had free access to water and standard chow. This study was performed in accordance with the recommendations in the Guide for the Care and Use of Laboratory animals of the National Institutes of Health. All experiments were approved by the Institutional Animal Care and Use Committee of Children's Hospital of Pittsburgh of UPMC.

**Figure 1:**
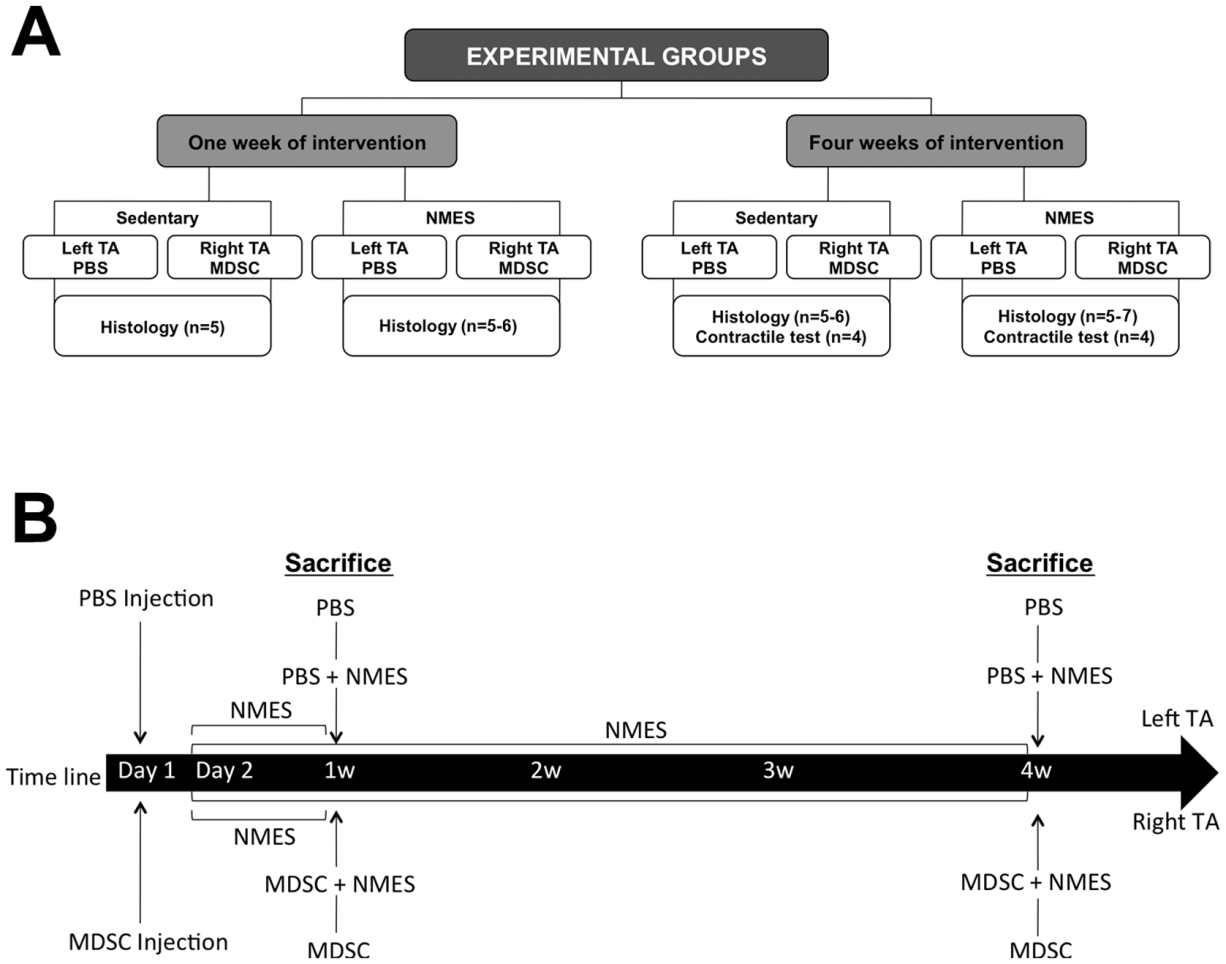
Schematic representation of the experimental groups and study design. (A) Schematic representation of the experimental groups. All interventions were performed for 1 or 4 weeks. For each time point, animals were randomly divided into 2 groups: Sedentary and/or NMES. For all animals, the left TA was injected with PBS and the right TA injected with MDSC. Muscles were collected for histological (1 and 4 weeks) and contractile analyses (4 week only). (B) Schematic representation of the experimental design.

Animals were randomly divided into the following groups: Saline controls (PBS, n = 14 muscles), Muscle-Derived Stem Cell injection (MDSC; n = 16 muscles), Saline + Neuromuscular Electrical Stimulation (PBS+NMES; n = 16 muscles) and MDSC+NMES (n = 18 muscles). The NMES interventions were performed for 1 or 4 weeks, allowing for the investigation of early and late skeletal muscle responses to MDSC transplantation and NMES. The muscles collected were evaluated using histology (n = 5–7 muscles/group) and functional testing (n = 4 muscles/group)(Figure 1A).

For MDSC transplantation, NMES, and animal sacrifice, mice were first anesthetized using 2% isofluorane, administered by inhalation. Following completion of the experimental procedures, the animals were sacrificed by cervical dislocation.

### Cells transduction to express LacZ reporter gene

MDSC were genetically engineered to express nuclear-localized LacZ (gift from Dr. P. Robbins, University of Pittsburgh) reporter gene to enable tracking of cells after transplantation. Cells were transduced with the retroviral vector MFG-NB containing a modified LacZ gene, which includes a nuclear-localization sequence cloned from the simian virus 40 (SV40) large tumor antigen, and is transcribed from the long terminal repeat. Prior to injection, transduced MDSC were assayed for LacZ expression, as described previously [16]; 50% of the MDSC population was positive for LacZ expression immediately prior to injection.

### Wild type MDSCs transplantation into dystrophic skeletal muscle

On the day of injection, MDSCs were trypsinized and spun at 2000 rpm for 5 minutes. The resulting pellet was re-suspended in phosphate buffered saline (PBS)+0.1% microsphere beads solution.

Bilateral anterior lower limbs were shaved. Animals in the MDSC and MDSC+NMES groups received a single intramuscular injection at the mid-belly (region that has the greatest muscle girth) of the right tibialis anterior (TA) containing 1.0×10^5^ MDSCs suspended in 20 µl of phosphate buffer saline (PBS)+0.1% fluorescent microsphere bead solution. For PBS and PBS+NMES groups, left side TAs served as controls, and were injected with an equal volume of PBS+0.1% fluorescent microsphere beads (Figure 1 A–B). Fluorescent microsphere beads were used in order to localize the injection site at the time of histological analysis.

### Neuromuscular Electrical Stimulation (NMES)

To investigate the ability of muscle contractile activity to dictate donor cell behavior *in vivo*, NMES was performed using a Neuromuscular Stimulator (Empi 300 PV, St Paul, USA) and modified surface electrodes, as previously described [17]. Prior to stimulation, the anterior lower limb was shaved. Location of the peroneal nerve was confirmed when stimulation resulted in a full hindlimb dorsiflexion and digit extension, indicating stimulation of the anterior compartment muscles, including the TA and extensor digitorum longus (EDL) muscles.

The NMES protocol was initiated 24 hours after MDSC or PBS injection and was performed 5 days/week for 1 or 4 weeks. The NMES protocol consisted of 2 sets of 10 contractions. The parameters used to stimulate the muscles were: pulse duration of 150µs, frequency of 50 Hz, time on: 5 seconds, time off: 10 seconds, 0.5-second ramp and 0.5-second ramp down. NMES intensity started at 9.0 mA and was gradually increased throughout the training, to a maximal intensity of 18.0 mA.

### Functional testing

Animals submitted to a 4-week intervention were subjected to a contractile testing of the lower leg anterior compartment muscles (TA and EDL) before their muscles were collected for histological analysis. We did not perform contractile this analysis in the 1-week treated animals since we did not expect functional improvements to appear so soon after cell transplantation or NMES interventions. The contractile testing was performed using an *in situ* testing apparatus (Model 809B, Aurora Scientific Inc, Canada), a stimulator (Model 701C, Aurora Scientific Inc, Canada), and a force transducer (Aurora Scientific Inc, Canada). The method used allows for the determination of muscle contractile properties of a muscle of interest, while maintaining normal muscle orientation, innervation and vascular supply. Briefly, the peroneal nerve of anesthetized animals was isolated through a small incision lateral to the knee. Mice were then placed supine on a 37°C heated platform and the foot being tested was positioned on the footplate. The hindlimb used for testing was stabilized with cloth tape on the knee and foot. Muscles were stimulated through the peroneal nerve by needle electrodes inserted beneath the skin.

Muscle peak twitch, time to peak twitch and half-relaxation time were evaluated with the ankle positioned at 20° of plantarflexion, the position which we determined to result in the greatest force output (data not shown). Tetanic contractions at 10, 30, 50, 80, 100, 120, 150 Hz were elicited to obtain a force-frequency curve, with a 2-minute rest between each contraction. The muscles were then subjected to a 7-minute long high-frequency fatigue protocol consisting of a series of short 350 ms tetanic contractions at 100 Hz, with 4-second intervals between contractions [18]. Using this protocol, muscles were stimulated a total of 105 times during the course of the protocol. Force recovery was analyzed at 5 and 10 min following completion of the fatiguing protocol. All surgical procedures and data collection were performed by the same blinded researcher. Results of single stimulations, tetanic contraction, fatigue and recovery from fatigue were collected in torque (milliNm). From torque measurements, the specific muscle force was obtained by normalizing the absolute force values (milliN) to the calculated muscle cross-sectional area (CSA). CSA was calculated as the muscle weight (mg)/muscle length (mm)×muscle density of 1.06 mg/mm^3^ [19]. Fatigue and force recovery data (5 and 10 min) were also expressed as a percentage of the maximum tetanic force event measured on the first contraction of the fatiguing protocol.

### Histological analysis

After 1 or 4 weeks, animals were sacrificed, and the TA muscles were harvested and immediately frozen in 2 methyl-butane pre-cooled in liquid nitrogen and stored at −80°C. Serial cross-sections (10 µm) were obtained and mounted onto slides. Immunofluorescent staining for dystrophin positive fibers (dys+), laminin, CD31 positive cells (as a measure of muscle capillarity), and embryonic myosin heavy chain (eMHC) were performed. Additionally, a Hematoxylin & Eosin stain was performed in order to determine the extent of fiber regeneration and damage, LacZ staining was performed to identify transplanted cells, and Masson's Trichrome stain was performed to quantify collagen content. All histological images were photographed and analyzed by a researcher blinded to the animal grouping.

#### Immunofluorescent staining

Dystrophin, laminin, and CD31: Sections were washed with PBS, fixed in 4% formalin for five minutes, and washed twice in PBS. Nonspecific binding was then blocked for 1 hour using PBS with 10% Donkey Serum (DS) or 5% Horse Serum (HS), for dystrophin and laminin, or for CD31 stains, respectively. Afterward, sections were incubated for 1 hour at room temperature with either a rabbit anti-mouse polyclonal dystrophin antibody (1∶300 dilution in 10% DS), rat anti-mouse monoclonal laminin antibody (1∶300 dilution in 10% DS), or with a rat anti-mouse primary antibody (1∶300 dilution in 5% HS) for CD31 staining. Following 3 PBS washes, anti-dystrophin sections were treated for 1 hour with a Cy3-labeled donkey anti-rabbit secondary antibody (1∶200 dilution in 10% DS); anti-laminin sections were treated for 1 hour with an anti-rat secondary antibody (1∶200 dilution in 10% DS); and anti-CD31 sections were treated for 1 hour with a 555-labeled goat anti-rat secondary antibody (1∶300 dilution in 5% HS). Samples were washed again 3 times in PBS and incubated with the nuclear stain 4'-6-Diamidino-2-phenylindole, DAPI, (1∶1000 dilution in PBS).

Embryonic myosin heavy chain: Sections were fixed in 100% cold methanol at room temperature for five minutes, washed once in PBS, and incubated with Avidin and Biotin (30 min each). Mouse on mouse (M.O.M) kit (Vector, BMK-2202) was used according to manufacturer instructions. Sections were incubated overnight at 4° with anti-mouse eMHC antibody (Developmental Studies Hybridoma Bank, University of Iowa; 1∶200 dilution in M.O.M diluent). Sections were then treated for 1 hour at room temperature with streptavidin anti-mouse (1∶200 dilution in M.O.M diluent). Samples were washed 2 times at 2 minutes with PBS and incubated with DAPI, (1∶1000 dilution in PBS) for 7 minutes.

#### Quantification of dystrophin positive myofibers and vascularity

To quantify the total number of dystrophin positive (dys+) fibers, the entire section containing the most fluorescent beads was identified and photographed using fluorescent microscopy (Nikon Eclipse E800; Japan; 20× objective). The total number of dys+ was manually counted using Northern Eclipse Software (Empix Imaging Inc.). Similarly, the number of capillaries was quantified as the total number of CD31 positive cells in the section containing the most beads. For the MDSC, PBS+NMES and MDSC+NMES groups, CD31 results are expressed as fold change compared to PBS control.

#### Quantification of eMHC positive fibers, myofiber regeneration and cross-sectional area (CSA)

The total number of eMHC positive fibers in the section containing the most beads was manually counted using NIS-Elements software (40× magnification). Additionally, 2 random pictures within the same section were obtained (40× objective) using a Nikon 90i motorized upright fluorescent microscopy and the average cross-sectional area (CSA) of the eMHC positive myofibers were evaluated using NIS-Elements software. Hematoxylin and Eosin (H&E) stains were performed in order to quantify the regeneration index (RI) (total number of centrally located nuclei/total number of fibers) for each experimental group, as previously described [8]. For each sample, the section containing the most fluorescent beads was selected and 3 random pictures were obtained (20× objective) using a light microscope (Nikon Eclipse E800; Nikon, Japan). The total number of fibers and the number of fibers with centrally located nuclei were manually counted using the National Institutes of Health (NIH)–developed image analysis software, Image J.

Laminin stains were used to quantify the CSA of the host myofibers. The entire section containing the most fluorescent beads was selected and photographed (20× magnification), and reconstructed using Nikon 90i motorized upright fluorescent microscopy and NIS-Elements software. A total of 500 fibers per section were randomly selected and evaluated using Image J. After CSA analysis, the 500 myofibers evaluated were subdivided in quartiles according to size and classified as Small, Medium, Large or Extra large. For each section, the percent of fibers in each one of the size categories (small, medium, large, and extra large) was calculated and compared across groups.

#### Quantification of collagenous tissue

Masson's Trichrome staining was performed to quantify the percent of collagen content and muscle fiber in a muscle section. Slides were processed using Masson's Trichrome Stain Kit as per manufacturer's instructions (K7228; IMEB, Chicago, IL). This process stains skeletal muscle fibers red, collagen blue, and nuclei black. For each sample, the section containing the most fluorescent beads was selected and photographed using Nikon 90i motorized upright fluorescent microscopy and NIS-Elements. The percentage of the total collagen-positive area relative to the total cross-sectional area was calculated. For determination of collagen content, sections were analyzed using MetaMorph Offline.

#### Transplanted MDSC tracking through LacZ detection

Muscle sections were fixed with 1% glutaraldehyde (Sigma Chemical, St. Louis, MO) and were incubated with LacZ staining solution (0.5M K4Fe[CN]6, 0.5M K3Fe[CN]6, and 1.0M MgCl2) for 2 h at 37°C, as previously described [20]. The sections were subsequently stained with eosin.

For the muscle samples transplanted with MDSC, the total number of LacZ-positive cells within a muscle section was manually counted (10× objective) using ImageJ. A total of 15 muscle sections (200 µm apart), representing about 25% of the total muscle length (3 mm), were analyzed. In addition, in order to determine the percentage of LacZ-positive cells that were terminally differentiated towards a myogenic lineage, co-localization of LacZ and laminin was performed. The number of LacZ-positive cells contained clearly within the borders of a myofiber (defined by Laminin stain) was manually counted using a 20× objective, divided by the total number of LacZ-positive cells within the field of view, and expressed as a percentage.

### Statistics

All analyses were performed using standard statistical software packages (SPSS v17.0 software, Chicago, IL). Results are expressed as mean ± standard deviation. Shapiro-Wilk test was performed to assess normality of data, and Levene's test was used to check equality of variances. One-Way ANOVA and Kruskal-Wallis tests were used to compare differences across groups, with subsequent *post-hoc* Tukey and Mann-Whitney U tests, as appropriate. Independent t-test was performed to compare 1- and 4-week results within groups, as well as to compare LacZ results between MDSC and MDSC+NMES groups. To investigate the relationship between vascularity and the number of engrafted donor cells, a Pearson correlation was performed. Multivariate ANOVA was used to evaluate CSA of the host myofibers. One animal presented a score (total number of LacZ-positive cells within a muscle section) that was greater than 2 standard deviations outside the mean. Therefore, he was considered an outlier and was not included in the LacZ analysis. Statistical significance was established, *a priori*, at p≤0.05.

## Results

### Four weeks of NMES increases the number of CD31 positive cells

**Figure 2:**
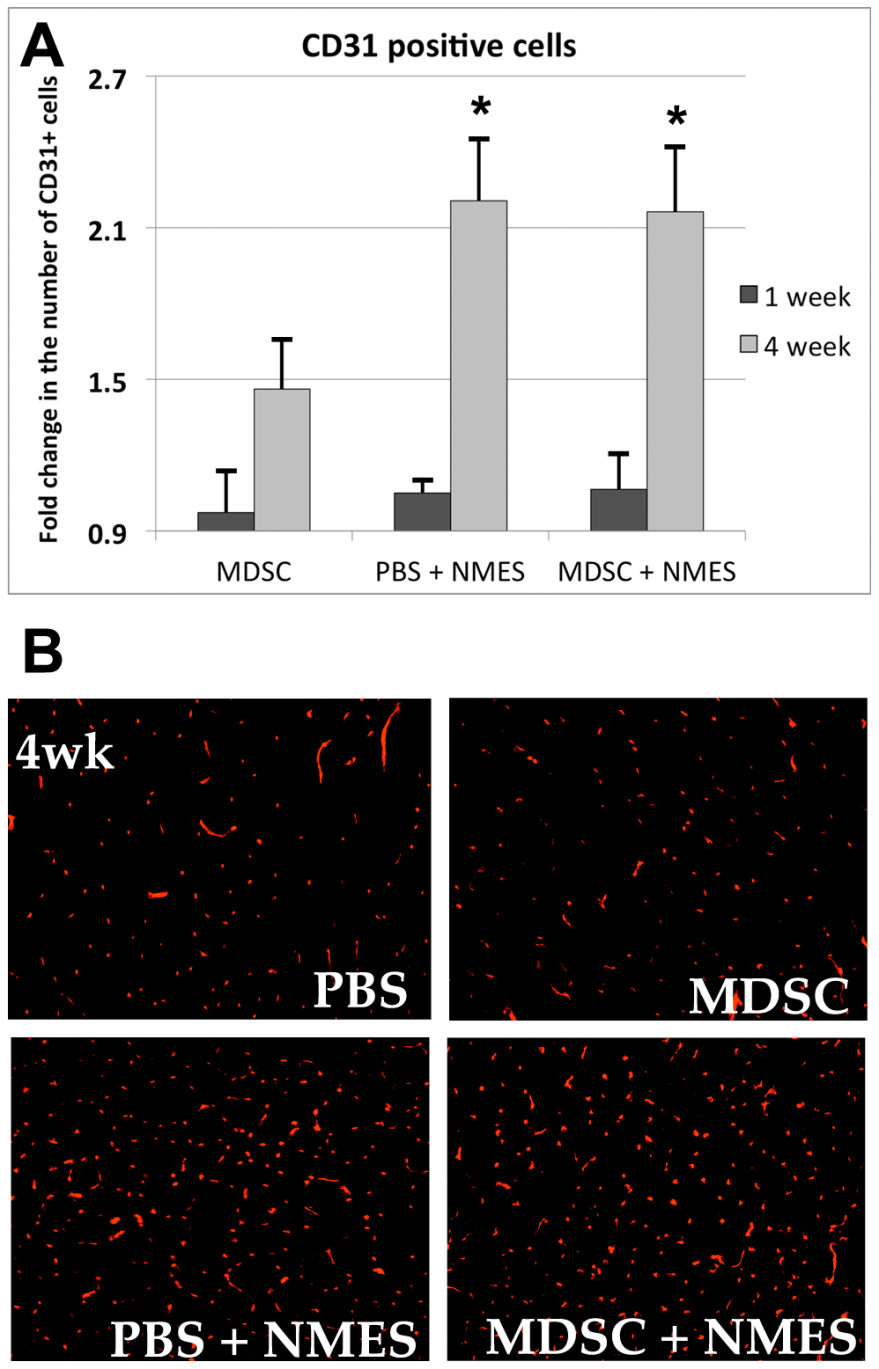
Effect of *in vivo* MDSC transplantation and NMES on CD31 positive cells in dystrophic muscles. (A) Fold change in CD31+ cells on MDSC, PBS+NMES and MDSC+NMES groups, when compared to PBS injected controls (n = 5–6/group). (B) CD31 immunostaining in the tissue cross-sections of TA muscles across experimental groups (20× magnification). * Denotes significantly different when compared to MDSC 4 week (p<0.05).

No differences were observed in the total number of CD31 positive cells between PBS and MDSC groups, at either 1 or 4 weeks after transplantation (PBS 1W: 1; MDSC 1w: 0.97±0.16; p = 0.602; PBS 4w: 1; MDSC 4w: 1.33±0.19; p = 0.286; figure 2A–B), suggesting that, for the dose and timing protocol used in this experiment, MDSC transplantation alone was not sufficient to significantly stimulate angiogenesis.

Similarly, one week of NMES did not promote significant changes in vascularity, as evidenced by the number of CD31 positive cells (PBS: 1; PBS+NMES: 1.05±0.05; MDSC+NMES: 1.06±0.14; p>0.273; figure 2A–B). On the other hand, 4 weeks of NMES resulted in a significant fold increase in the CD31 positive cells in the PBS+NMES and MDSC+NMES groups when compared to PBS and MDSC only groups (PBS: 1; MDSC: 1.33±0.19; PBS+NMES: 2.20±0.24; MDSC+NMES: 2.16±0.26; p<0.001; figure 2A–B).

### Four weeks of NMES increases the myogenic differentiation of transplanted MDSC

**Figure 3:**
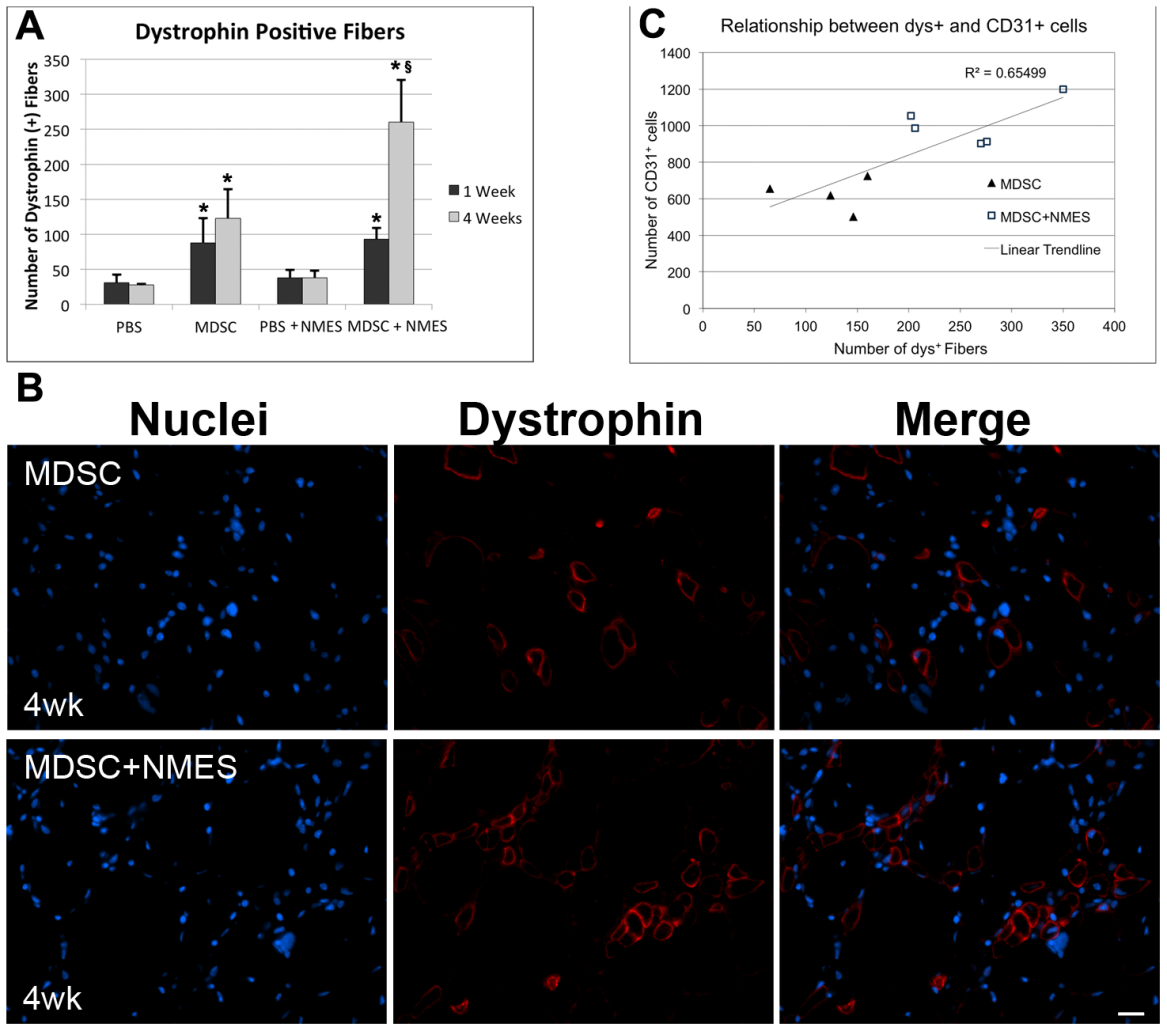
Effect of *in vivo* MDSC transplantation and NMES on the number of dystrophin positive fibers in dystrophic muscles. (A) Total number of dys+ in each of the experimental groups (n = 5–6/group). (B) Dystrophin immunostaining in the tissue cross-sections of TA muscles of *mdx* mice 4 weeks after transplantation (40× magnification). (C) Correlation between the number of dys+ and CD31+ cells across our 4week transplantation groups (R^2^ = 0.655; p = 0.004; n = 9). * Denotes significantly different when compared to PBS counterparts control (p<0.05). ^§^ Denotes significantly different when compared to MDSC 4-week group (p<0.05).

Since dystrophic animals largely lack dystrophin expression, quantification of the number of dys+ fibers present in the host after MDSC transplantation is an accepted method to evaluate the myogenic potential of donor MDSC. As expected, a significant increase in the number of dys+ fibers was observed in the MDSC group when compared to PBS group both 1 and 4 weeks after transplantation, (PBS 1w: 31±11; MDSC 1w: 88±35; p = 0.002; PBS 4w: 28±1; MDSC 4w: 123±41; p = 0.034; figure 3), confirming the potential of donor cells to differentiate towards a myogenic lineage. However, no significant differences were found in the total number of dys+ fibers in the MDSC groups between 1 and 4 weeks (p = 0.215).

On the other hand, 4 weeks of NMES following MDSC transplantation resulted in a 2-fold increase in the number of dys+ fibers when compared to MDSC control group (MDSC: 123±41; MDSC+NMES: 260±60; p = 0.014; figure 3A–B), suggesting that NMES significantly increased the myogenic differentiation of transplanted MDSCs. Moreover, while there was no relationship between muscle vascularity and the number of dys+ fibers after 1 week (R^2^ = 0.0121; p = 0.374) we did observe a significant positive correlation between the number of dys+ and CD31+ cells across our 4-week transplantation groups (R^2^ = 0.655; p = 0.004) (Figure 3C).

**Figure 4:**
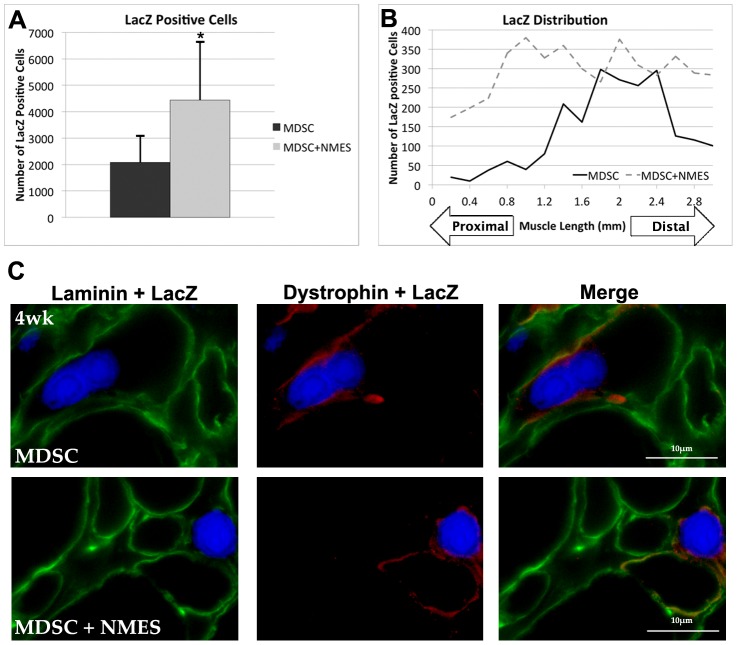
LacZ positive cells among experimental groups. (A) Total number of LacZ positive cells (B) LacZ distribution in the MDSC and MDSC+NMES 4-week groups across 4 mm of the muscle (n = 6–7/group). (C) LacZ, laminin and dystrophin staining in the tissue cross-sections of TA muscles in MDSC and MDSC+NMES 4-week groups (60× magnification, scale bar = 10 µm). * Denotes significantly different when compared to PBS 1-week control (p<0.05). § Denotes significantly different when compared to PBS 4-week group (p<0.05).

Given the increased number of dys+ fibers following NMES as compared to control counterparts at the later timepoint, we then compared the total number of LacZ-positive cells across a total distance of 3 mm in the MDSC and MDSC+NMES groups. After 4 weeks, there was a significant increase in the total number of LacZ-positive cells following completion of an NMES protocol, when compared to MDSC transplantation alone (MDSC: 2079±1006; MDSC+NMES: 4438±2185; p = 0.03; figure 4A–B).

Additionally, approximately 64% and 77% of the LacZ cells were co-localized within myofibers for both MDSC and MDSC+NMES groups, respectively (Figure 4C). The remainder of the LacZ-positive cells appeared to be localized to the cellular infiltrate at the transplantation site. To investigate whether the use of NMES resulted in larger LacZ positive myofibers we also evaluated the cross-sectional area of the LacZ positive fibers. No differences were observed in the CSA of LacZ positive myofibers when comparing across MDSC and MDSC+NMES groups (MDSC: 757.83±153.88 µm; MDSC+NMES: 774.48±181.39 µm; p = 0.893).

### Four weeks of NMES increases the number, but not the CSA, of eMHC muscle fibers

A significant increase in the number of eMHC myofibers was observed in the MDSC group when compared to PBS group, both 1 and 4 weeks after transplantation (PBS 1w: 94±146; MDSC 1w: 496±136; p = 0.007; PBS 4w: 126±131; MDSC 4w: 852±275; p = 0.004). Despite the fact that no significant differences were observed between MDSC and MDSC+NMES groups 1 week after transplantation (MDSC 1w: 496±136; MDSC+NMES 1w: 532±230; p = 0.983), animals submitted to 4 weeks of NMES presented an increased number of eMHC positive myofibers when compared to MDSC (MDSC 4w: 852±275; MDSC+NMES 4w: 1351±387; p = 0.016).

No differences were observed in the CSA of eMHC positive myofibers between the groups either 1 (PBS 1w: 181±71; MDSC 1w: 152±49; PBS+NMES 1w: 115±22; MDSC+NMES 1w: 148±45; p = 0.278) or 4 weeks (PBS 4w: 183±71; MDSC 4w: 171±29; PBS+NMES 4w: 170±44; MDSC+NMES 4w: 146±36; p = 0.489) after transplantation.

### Muscle regeneration, myofiber CSA and fibrosis formation were not influenced by NMES

**Figure 5:**
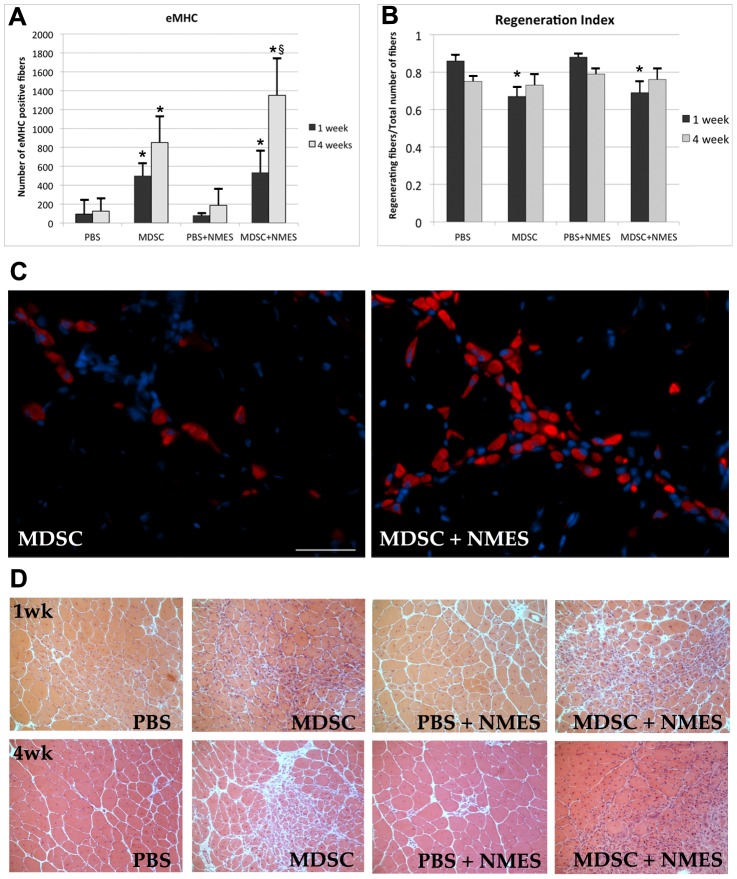
Effect of *in vivo* MDSC transplantation and NMES on muscle regeneration. (A) Total number of eMHC positive myofiber/section in each experimental group (n = 5–6/group). (B) Regeneration index (total number of regenerating fibers/total number of fibers) between experimental groups at 1 and 4 weeks (n = 5–6/group). (C) eMHC staining in the tissue cross-sections of TA muscles of *mdx* mice 4 weeks after MDSC transplantation (40× magnification, scale bar = 20 µm). (D) Hematoxylin & Eosin staining in the tissue cross-sections of TA muscles of *mdx* mice 1 and 4 weeks after transplantation (20× magnification). * Denotes significantly different when compared to PBS 1-week control (p<0.05).

One week after MDSC transplantation, there was a significant decrease in the regeneration index of both MDSC and MDSC+NMES groups, when compared to PBS and PBS+NMES controls (MDSC: 0.67±0.05; MDSC+NMES: 0.69±0.06; PBS:0.86±0.03; PBS+NMES: 0.88±0.01; p<0.01; Figure 5). Animals transplanted with MDSC showed a non- significant increase in the total number of muscle fibers per field analyzed (p = 0.463), as well as a non-significant decrease in the total number of fibers with central nuclei (p = 0.068), therefore leading to a significant decrease in the regeneration index (total number of centrally nucleated fibers/total number of fibers). However, 4 weeks after MDSC transplantation, no differences were observed in the regeneration index of the groups (Figure 5). These findings may suggest that, early after transplantation, donor cells may interfere with the degeneration/regeneration cycles typical of dystrophic muscle, but this effect is not sustained at later time points.

**Figure 6:**
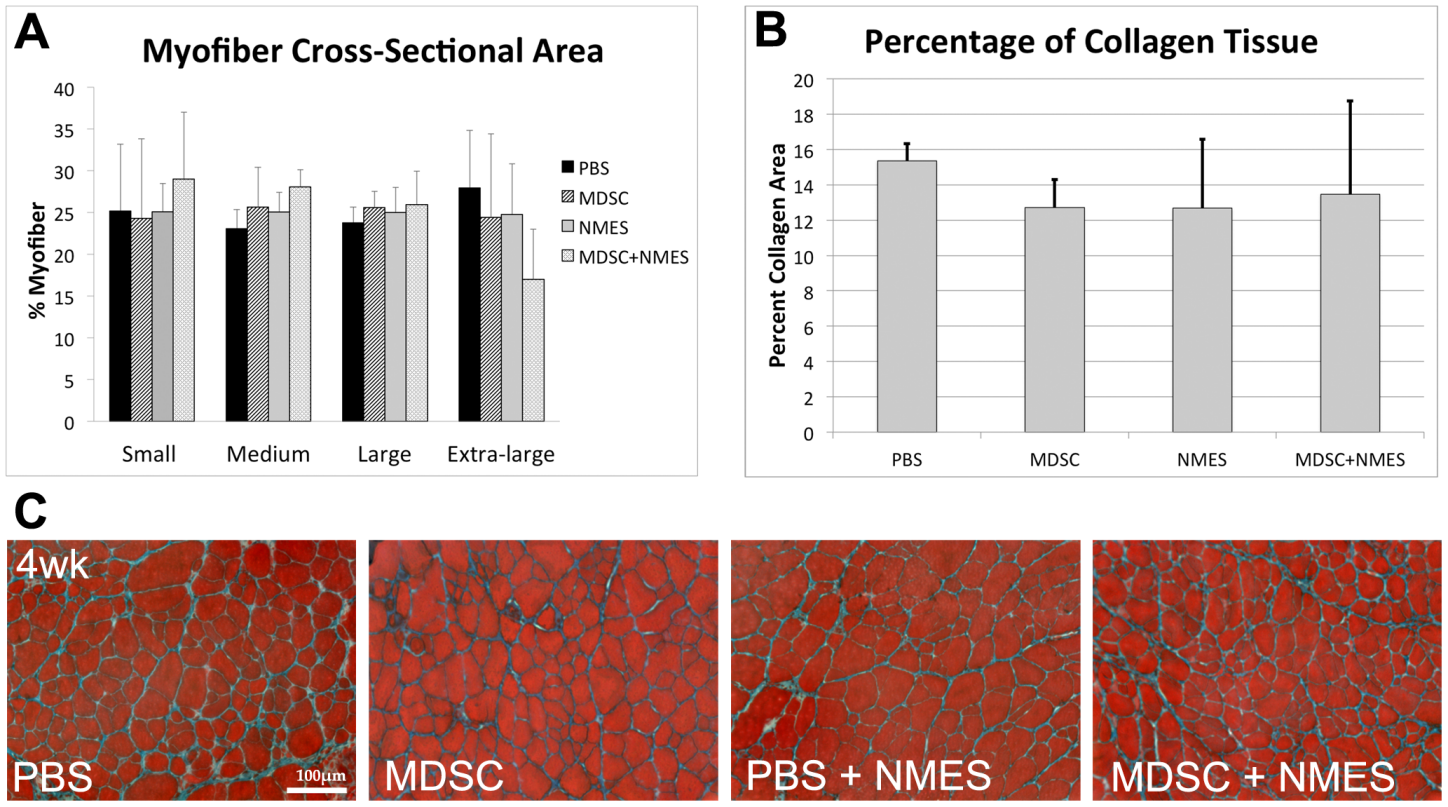
Effect of in vivo MDSC transplantation and NMES on cross-sectional area of host myofibers and percent of collagenous tissue. (A) Distribution of myofibers in each group classified as ‘small’, ‘medium’, ‘large’ and ‘extra-large’, expressed as percentage. (B) Percent of collagenous tissue across experimental groups. (C) Masson's Trichrome stain in the tissue cross-sections of TA muscles of mdx mice 4 weeks after transplantation (20× magnification, scale bar = 100 µm).

Four weeks after MDSC transplantation and/or NMES, no differences were observed in the distribution of ‘Small’, ‘Medium’, ‘Large’, or ‘Extra-large’ fibers (Small, p = 0.819; Medium, p = 0.206; Large, p = 0.714; Extra-large, p = 0.250; figure 6A). Similarly, no differences in the percentage of collagenous tissue (PBS: 15.36±0.96; MDSC: 12.70±1.6; NMES: 12.68±3.9; MDSC+NMES: 13.46±5.29; p = 0.567; Figure 6B–C) were observed between the groups.

### NMES and/or the combination of MDSC and NMES improve contractile characteristics

Contractile testing showed no differences in maximal twitch torque (p = 0.053), specific twitch force (p = 0.540), time to peak twitch (p = 0.627), or half relaxation time (p = 0.432) between groups (Table 1).

**Table 1:** Contractile characteristics.

	PBS	MDSC	NMES	MDSC+NMES
Mice (*n*)	4	4	4	4
Muscle weight (mg)	80.6±3.47	69.4±4.0	72±9.6	63.4±13
Muscle length (mm)	11.57±0.06	11.49±0.06	11.66±0.26	11.38±0.27
CSA (mm^2^)	6.57±0.31	5.7±0.34	5.8±0.69	5.2±0.95
Maximum twitch torque (MilliN m)	0.735±0.151	0.604±0.045	0.748±0.106	0.598±0.069
Maximum tetanic torque, 150 Hz (MilliN m)	2.24±0.39	2.34±0.32	2.66±0.39	2.33±0.05
Specific twitch force (MilliN/mm^2^)	3.71±0.67	3.54±0.30	4.28±0.14	3.98±1.28
Specific tetanic force, 150 Hz (MilliN/mm^2^)	11.30±1.50	13.76±1.95	15.22±0.89*	16.19±2.48*
Time to peak twitch (ms)	28±4.9	26±3.2	30±3.4	27±5.6
Half relaxation time (ms)	22±4.8	22±4.3	28±7.1	22±6.3

* Denotes significantly different when compared to PBS (p<0.05).

No differences were observed in the maximum tetanic torque between groups (150 Hz; p = 0.369), however, there was a significant increase in the specific tetanic force (150 Hz) in both the PBS+NMES and MDSC+NMES groups, when compared to PBS (PBS: 11.30±1.50 milliN/mm^2^; MDSC: 13.76±1.95 milliN/mm^2^; PBS+NMES: 15.22±0.89 milliN/mm^2^; MDSC+NMES: 16.19±2.48 milliN/mm^2^; p<0.036) (Table 1). No differences were found between PBS+NMES and MDSC+NMES groups (p = 0.881), suggesting that the transplanted cells did not contribute to the increased specific tetanic force at the time-point investigated.

**Figure 7:**
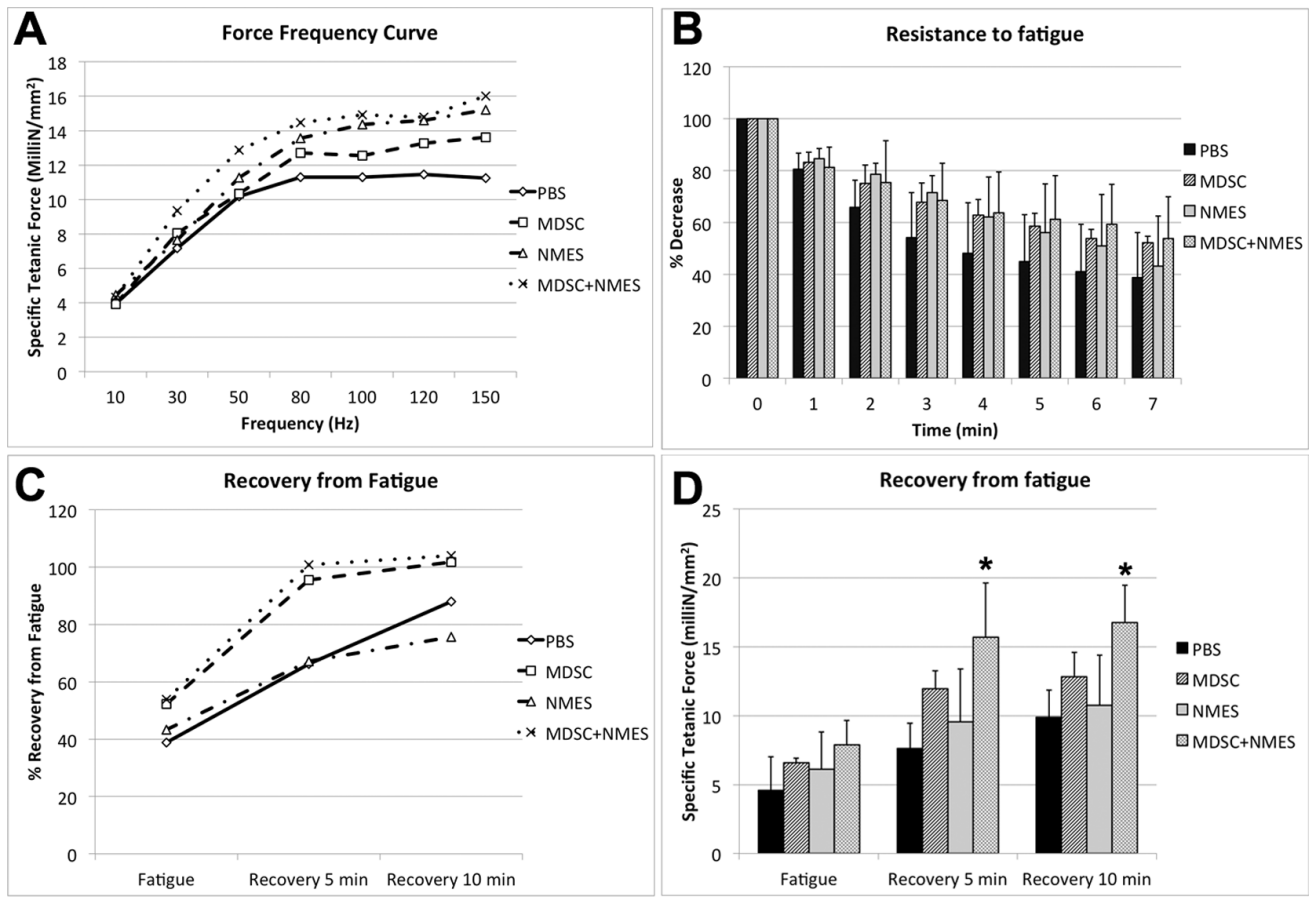
*In situ* contractile testing of the lower leg anterior compartment muscles (n = 4 per group). (A) Force-frequency curve. (B) Resistance to a 7-min fatigue protocol. Data are presented as percent force decrease compared to the first muscle contraction. (C) Recovery from fatigue after 5 and 10 minutes. Data are presented as percent force compared to the initial muscle contraction at the start of the fatigue protocol. (D) Recovery from fatigue after 5 and 10 minutes, expressed as specific tetanic force. * Different when compared to PBS and NMES groups (p<0.05).

While no differences were found in the resistance to fatigue between the groups (PBS: 38±17%; MDSC: 51±2.5%; PBS+NMES: 47±21%; MDSC+NMES: 46±11%, p = 0.507; figure 7B), muscles transplanted with MDSC demonstrated a non-significant trend to a faster recovery from fatigue, showing a near complete recovery from fatigue after 5 minutes of the end of the protocol when compared to PBS control (PBS: 67%±11%; MDSC: 96%±13%; NMES: 68%±26; MDSC+NMES: 93%±12%; p = 0.112) (Figure 7C).

Animals receiving a combination of both MDSC transplantation and NMES presented a significantly improved recovery from fatigue at both 5 (Specific tetanic force: PBS: 7.87±1.83 milliN/mm^2^; MDSC: 12.19±1.2 milliN/mm^2^; NMES: 9.63±3.9 milliN/mm^2^; MDSC+NMES: 15.96±4.14 milliN/mm^2^, p = 0.018) and 10 min (PBS: 10.13±2.07 milliN/mm^2^; MDSC: 13.11±1.81 milliN/mm^2^; NMES: 10.88±3.56 milliN/mm^2^; MDSC+NMES: 17±2.76 milliN/mm^2^, p = 0.025), when compared to PBS group, figure 7D.

## Discussion

The host microenvironment plays an essential role in determining transplanted cell fate, supporting the importance of optimizing the niche to maximize the efficacy of cellular therapies [2]–[4]. Given the chronic myofiber degeneration/regeneration cycles occurring within dystrophic muscles, and the replacement of healthy muscle into adipose and scar tissue, this represents a hostile environment for cell transplantation. Modulation of the microenvironment has the potential to provide muscle stems cells (both host and donor) with support factors necessary to promote survival and improve myogenic capacity. Our results suggest that wild type MDSC transplantation alone results in a limited cell engraftment and no significant increase in muscle strength. Conversely, the addition of NMES improves the myogenic potential of donor cells and enhances the peak tetanic strength of the dystrophic muscle. Only a combination therapy consisting of both cell transplantation and NMES resulted in a faster muscle recovery from fatigue when compared to controls.

Previous studies performed in our laboratory have demonstrated the ability of mechanical stimulation, such as functional overloading (which involves surgical ablation of the synergistic muscle) [7] and treadmill running [8], to improve MDSC engraftment following transplantation. Each of these methods, however, has their limitations. The ablation of a muscle to produce overloading is not a clinically desirable option and a high intensity exercise such as treadmill running may not be feasible in the case of myopathies or severe muscle injuries where overexertion may only exacerbate the pathology. NMES offers the advantage of being a widely used, safe, and inexpensive rehabilitation modality that may be applied in the case of many muscular diseases as well as for individuals not able to participate in exercise protocols.

In the current experiment, although 1 week of NMES did not affect donor cell engraftment, 4 weeks of NMES stimulated a significant increase in the myogenic differentiation of donor cells, as determined by the average number of dys+ myofibers/cross-section. Consistent with previous animal studies [7], [16], [21], the current findings also demonstrated a direct relationship between vascularity and donor cell engraftment (R^2^ = 0.655; p = 0.004). In a recent paper by Gargioli and colleagues [22], authors demonstrated an increased engraftment efficiency of tendon fibroblasts injected into aged dystrophic muscle when the fibroblasts were modified to express a pro-angiogenic factor. They hypothesized that donor cells survive better in a blood vessel-rich environment, supporting the role of the muscle microenvironment on stem cell transplantation efficiency. Of note, we observed no additive effect of NMES and MDSC transplantation on skeletal muscle vascularity, suggesting that the presence of transplanted MDSCs themselves did not significantly contribute to angiogenesis. The effects of NMES on muscle vascularity and the secretion of myogenically favorable growth factors, however, has been widely documented, further supporting its role as an adjunct therapy to cell transplantation [11], [23]. Future mechanistic studies should investigate whether targeted inhibition of NMES-induced angiogenesis, such as through the administration of sFLT1, a VEGF antagonist, would abrogate the beneficial effect of NMES on cell transplantation and muscle function.

In addition to the vascular changes observed, we found that the application of NMES, both in the presence and in the absence of donor cells, enhanced the specific tetanic force of dystrophic muscles when compared to PBS- or MDSC-injected muscles. These findings suggest that NMES may represent a clinically-relevant method to improve the muscle strength of dystrophic muscle. NMES has been shown to recruit motor units in a non-specific and temporally synchronous manner [24]. Therefore, the beneficial effect of NMES on force production may be attributed to increased neural activation, consistent with previous reports. This would also help explain why increases in force were not concomitant with an increased myofiber hypertrophy of NMES-treated muscles. However, we did not observe a further increase in skeletal muscle strength when NMES was coupled with MDSC transplantation. Qualitatively, it is clear that, even 4 weeks after transplantation, donor fibers are very small in diameter. It is possible that longer evaluation time points are necessary to allow for donor fibers to mature, hypertrophy, and more effectively contribute to an overall enhanced skeletal muscle function. Given the majority of the donor cells present within the muscle were still contained within small, nascent myotubes, it is likely that these nascent donor fibers are not yet innervated and, therefore, do not possess the capacity to contribute to functional improvements. To the best of our knowledge, no studies to date have investigated the innervation status of MDSC-derived fibers following transplantation. This should be the topic of future investigations.

Neither NMES nor stem cell transplantation, in isolation or in conjunction, had any effect on the fatigue resistance of dystrophic muscles. However, animals transplanted with MDSC did demonstrate a trend towards a faster recovery from fatigue after 4 weeks, when compared to control counterparts (Figure 7C), and both MDSC-treated groups (MDSC and MDSC+NMES) almost completely recovered to baseline strength after 5 minutes. However, only the combination of MDSC transplantation and NMES resulted in a significantly increased maximal tetanic force both 5 and 10 min after cessation of a fatiguing protocol.

It has been previously reported that dystrophic muscles demonstrate not only a decreased resistance to fatigue, but also an impaired fatigue recovery, as evidenced by a persistent muscle acidosis following a fatiguing protocol [25]. This impaired fatigue recovery implies a metabolic dysfunction of dystrophic muscles. Indeed, structures associated with proton expulsion following completion of a contractile protocol have been localized to the myofiber membrane, and therefore, dystrophin [25]. Our current results suggest that MDSC transplantation may help overcome this dysfunction by allowing the muscle to more rapidly return to baseline force-producing capacity. This finding is important considering fatigue is a prominent and common symptom for individuals with muscular dystrophy, and one that oftentimes limits their ability to participate in activities of daily living. Adequate physical functioning is reliant both on an individual's ability to produce a sufficient force to perform daily task and the individual's ability to sustain tasks over time. Therefore, improvements in a muscle's ability to recover from a fatiguing activity could ultimately translate to an enhanced quality of life. The mechanisms, however, underlying the MDSC-mediated hastened muscle recovery remain unclear. Further studies should be performed to better understand the metabolic dysregulation of dystrophic muscles following a fatiguing protocol, and as well as the capacity of transplanted cells to ameliorate these effects.

Interestingly, it was only when we administered both NMES and MDSC transplantation that we observed significant increases in both strength and fatigue recovery, as compared to controls. It appears that the application of NMES (independent of MDSC transplantation) increased the overall force-producing capacity of dystrophic muscle, whereas MDSC transplantation accelerated the muscle's capacity to return to maximal capacity after a fatigue protocol. These results suggest a synergistic effect of NMES and MDSC transplantation to promote an overall functional improvement of dystrophic muscle. As biological therapies are being translated to the clinic, increasing attention should be paid towards the development of comprehensive clinical care plans that will maximize the overall therapeutic benefit of the intervention. This involves not only optimization of stem cell transplantation protocols and methods to maximize transplantation efficiency, but also careful attention to the development of targeted and specific rehabilitation protocols designed to promote maximal muscle function.

A limitation of this study is that the analysis of contractile function and histological characteristics did not extend beyond 4 weeks. As such, it is unclear whether any beneficial effects of NMES or MDSC transplantation may persist, increase or decrease over time. We observed, for example, that 4 weeks after transplantation, approximately 30% of the transplanted cells were found outside of the muscle fibers. Since the LacZ nuclei are largely intact, we expect that the cells are still viable. However, it is not known whether these cells differentiated into fibrotic tissue [3] or if they are still undifferentiated. The observed increase in the dys+ and eMHC positive cells between 1 and 4 weeks suggests that at least a portion of the donor cells are still in a stage of proliferative expansion and are not yet differentiated.

An improved understanding of mechanisms underlying the beneficial effects observed in these experiments would aid in the optimization of NMES and transplantation protocols. The present experiment demonstrates a proof-of-principle that mechanical stimulation using NMES may be an effective method to dictate the behavior of donor cells, even after transplantation. Future studies should investigate whether other cell populations considered for cellular therapies are similarly responsive to NMES.

Taken together, our results suggest that modulation of the niche by NMES enhances both transplanted cell engraftment and strength of dystrophic muscle. Additionally, a combination therapy of NMES and MDSC results in a significant improvement in muscle force recovery following a fatigue protocol.
